# Emerging variants of methicillin-resistant *Staphylococcus aureus* genotypes in Kuwait hospitals

**DOI:** 10.1371/journal.pone.0195933

**Published:** 2018-04-18

**Authors:** Samar S. Boswihi, Edet E. Udo, Stefan Monecke, Bindu Mathew, Bobby Noronha, Tina Verghese, Sajida B. Tappa

**Affiliations:** 1 Department of Microbiology, Faculty of Medicine, Kuwait University. Jabriya, Kuwait; 2 Alere Technologies GmbH, Jena, Germany; 3 InfectoGnostics Research Campus, Jena, Germany; 4 Institute for Medical Microbiology and Hygiene, Technische Universität Dresden, Dresden, Germany; Rockefeller University, UNITED STATES

## Abstract

**Background:**

Frequent changes in the epidemiology of methicillin-resistant *Staphylococcus aureus* (MRSA) occurring worldwide demand regular surveillance to study their composition and distribution in healthcare facilities. We investigated the genotypic characteristics of MRSA obtained in Kuwait hospitals to better understand their clonal distribution.

**Materials and methods:**

A total of 1,327 MRSA isolates obtained from clinical samples in 13 Kuwait hospitals from 1 January to 31 December 2016 were investigated using antibiogram, SCC*mec* typing, spa typing and DNA microarray.

**Results:**

The isolates belonged to six SCC*mec* types with the majority belonging to type IV (658; 49.5%) and type V (355; 26.7%). Two hundred and sixty-one spa types were identified with spa types t688, t304, t860, t127, t044, t311, t002, t223, t267, t019, t3841, t005, t084, t852, and t657 constituting 51.0% (n = 677) of the isolates. Among the 1,327 MRSA isolates, 102 (7.68%) isolates were identified as novel variants of internationally recognized MRSA clones. These 102 isolates were investigated further and belonged to 14 clonal complexes (CCs) with CC361 (32; 32.3%), CC30 (15; 14.7%), CC22 (13; 12.7%) and CC1 (11, 10.7%) as the dominant CCs. Eighty-one (79.4%) of the novel isolates harbored SCC*mec* IV or V+*fusC* composite genetic elements. Four isolates (3.9%) harbored unusual combinations of *ccr* and *mec* complexes comprising of CC6-MRSA [IV+*fusC*+*ccrC*], CC97-MRSA [V/V_T_+*fusC*+*ccrAB2*], CC121-MRSA [V/V_T_+*fusC*+*ccrB4*] and CC1-MRSA-pseudoSCC*mec* [class B mec+*fusc*+*ccrAB1*]. Forty-six (45.1%) of these isolates were positive for PVL and 89 (87.2%) were resistant to fusidic acid mediated by *fusC*.

**Conclusions:**

The study showed the emergence of novel variants of previously recognized MRSA genotypes with unusual genetic characteristics including high prevalence of PVL and fusidic acid resistance in Kuwait hospitals. This has added to the dynamic lists of known variations in MRSA genomes which can impose serious challenges for infection control and treatment of MRSA infections.

## Background

Methicillin-resistant *Staphylococcus aureus* (MRSA) can spread rapidly in hospitals and other healthcare settings resulting in an increased workload for healthcare workers and economic burden [1]. Prior to the 1990s, MRSA was reported mostly in elderly patients admitted to healthcare facilities with previous history of hospitalization and antibiotic treatment. However, beginning in the early 1990s MRSA strains were reported in patients in the community with no previous history of admission to healthcare facilities in Western Australia and elsewhere [2, 3]. These strains were different from the multiply-resistant epidemic MRSA (EMRSA) that were prevalent at the time by being susceptible to most non -beta-lactam antibiotics and were subsequently designated non-multiresistant MRSA or community-originated / community-associated MRSA (CA-MRSA) [3, 4]. One of the early CA-MRSA strains reported belonged to ST30, a clone known as the Southwest Pacific clone (SWP) [5]. Since then, several CA-MRSA belonging to diverse clones have been reported worldwide [6, 7].

The composition of MRSA clones is changing in different healthcare facilities in different countries. For example, the CA-MRSA clone, ST30-MRSA-IV replaced the multiresistant clone, ST239-MRSA-III, in Singapore and Malaysian hospitals [8]. Studies from the United Arab Emirates [9], Portugal [10], India [11] and Germany [12] have also reported the replacement of ST239-III-MRSA by CA-MRSA clones. Similarly, ST22-MRSA-IV and ST772-MRSA-V have replaced ST239-MRSA-III as the dominant clones in Indian hospitals [13].

Furthermore, a recent study which investigated changes in the epidemiology of MRSA strains from 1992 to 2010 in Kuwait hospitals revealed that most of the MRSA strains obtained in the 1990s belonged to the well-studied healthcare -associated MRSA genotype, ST239-MRSA-III. However, since 2010 the prevalence of ST239-MRSA-III strains declined accompanied by an increase in the number and diversity of CA-MRSA clones including ST772, a clone widely reported in India and Bangladesh and in other countries [14].

In furtherance of the need to obtain current data on the epidemiology of MRSA strains in Kuwait hospitals, MRSA obtained from1 January to 31 December 2016 were investigated using a combination of molecular typing techniques. Results of molecular typing revealed the presence of a mixed population of MRSA composed of internationally recognized clones and variants of those clones that were not previously reported in Kuwait. The purpose of this study was to characterize the novel variants of these MRSA clones for antimicrobial resistance and carriage of virulence-related genes.

## Materials and methods

### Bacterial strains

The MRSA isolates used in this study were collected as part of routine diagnostic microbiology investigations and later submitted to the MRSA Reference Laboratory for molecular typing. In total, 1,327 MRSA isolates collected from 13 different hospitals in Kuwait from 1 January to 31 December 2016 were investigated. Isolates were obtained from clinical samples collected from patients and identified using standard bacteriological methods including growth on Mannitol Salt Agar, Gram stain, tube coagulase and DNase testing. Isolates were preserved in 40% glycerol (v/v) in brain heart infusion broth at -80°C. Isolates were subcultured twice on brain-heart infusion agar (BHIA) before processing.

### Antibiotic susceptibility testing

Antibiotic susceptibility testing was performed by disc diffusion method according to the Clinical Laboratory Standards Institute (CLSI) [15] with the following antimicrobial disks (Oxoid, Basingstoke, UK): benzylpenicillin (2U), cefoxitin (30 μg), kanamycin (30 μg), mupirocin (200 μg and 5 μg), gentamicin (10 μg), erythromycin (15 μg), clindamycin (2 μg), chloramphenicol (30 μg), tetracycline (10 μg), trimethoprim (2.5 μg), fusidic acid (10 μg), rifampicin (5 μg), ciprofloxacin (5 μg), teicoplanin (30 μg), and linezolid (30 μg). Susceptibility to tested antibiotics was interpreted according to the CLSI. Minimum inhibitory concentration (MIC) for cefoxitin, vancomycin, teicoplanin and mupirocin were determined with Etest strips (AB BioMerieux, Marcy l’Etoile, France) according to the manufacturer's instructions. *S*. *aureus* strain ATCC25923 was used as a quality control strain for susceptibility testing. The D-test was used to test for inducible resistance to clindamycin. Susceptibility to fusidic acid was interpreted according to the British Society to Antimicrobial Chemotherapy [16].

### Molecular typing

SCC*mec* typing was performed on all MRSA isolates as described previously [17]. Spa typing was performed using protocol and primers published previously [18]. DNA for PCR amplification was isolated and purified as described previously [19].

### DNA microarray

The 1,327 MRSA isolates representing each spa type detected in each hospital were selected for DNA microarray analysis. The *S*. *aureus* genotyping Kit 2.0 (Alere Technology, Jena, Germany) was used following protocols provided by the manufacturer [7]. DNA microarray was performed to assign the MRSA isolates to clonal complex (CC). The method detects 334 target sequences including genes encoding species markers, SCC*mec*, capsule and agr group typing markers, exotoxins, adhesins, and antibiotic resistance [7].

## Results

The 1,327 MRSA isolates investigated in this study were analyzed using antibiotic susceptibility testing, SCC*mec* typing and spa typing. Antibiotic susceptibility testing showed that all MRSA isolates were susceptible to vancomycin, teicoplanin and linezolid. Besides beta-lactam resistance, resistance was observed for fusidic acid (619; 46.6%), kanamycin (563; 42.4%), erythromycin and clindamycin (521;39.2%), trimethoprim (521; 39.2%), ciprofloxacin (508; 38.3%), tetracycline (427; 32.1%), chloramphenicol (120; 9.0%), and high-level mupirocin (49; 3.7%). Fewer isolates were resistant to rifampicin (3; 0.2%). In total, 658 (49.5%) of the MRSA carried SCC*mec* type IV genetic element. This was followed by SCC*mec* type V (355; 26.7%), and type III (117; 8.8%). SCC*mec* type VI was detected in 112 (8.4%) of the isolates. SCC*mec* types I and II were detected in one (0.07%) and 13 (0.97%) isolates, respectively. Spa typing revealed that the isolates belonged to 261 spa types with spa types t688, t304, t860, t127, t044, t311, t002, t223, t267, t019, t3841, t005, t084, t852, and t657 constituting 51.0% (N = 677) of the isolates. A total of 102 MRSA strains were identified as novel variants of known genotypes that were previously reported in Kuwait hospitals and elsewhere [7, 14]. In this paper, only the 102 novel variants were investigated further. The novel variants were classified into 14 clonal complexes (CCs) and a singleton ST2867. A high proportion of the novel variants belonged to CC361 (32; 32.3%) followed by CC30 (15; 14.7%), CC22 (13; 12.7%) and CC1 (11, 10.7%). CC8, CC121 and CC152 were each detected in six (5.8) isolates. CC913 was detected in three (2.9%) isolates. CC45, CC88 and CC97 were each detected in two (1.9%) isolates, while CC5, CC6, CC1153, and the singleton ST2867 occurred in single (0.98%) isolates. Most of the novel variants carried *fusC* (89; 87.2%). Genes for PVL were detected in 46 (45.1%) of the isolates while the toxic shock syndrome toxin-1 gene, *tst1*, was detected in 24 (23.5%) isolates. The molecular characteristics of the novel variants are shown in Table 1.

**Table 1:** Virulence and antibiotic resistance encoding genes of the novel MRSA variants.

**CC**	**Novel MRSA variants (N)**	**Spa types (N)**	**Antibiogram**	**SCC*mec*-associated markers**	**Antibiotic resistance genes**	**Toxin genes**	**Miscellaneous**
CC1	CC1-MRSA-[V/V_T_+*fusC*] (PVL^+^) (8)	t127 (2), t2207 (6)	GM (2), K (6), E (5), CC (5), TE (5), FD (8)	*ugpQ*, *mecA*, *fusC*, *“ccrAA”*, *ccrC*	*ermC*, *aacA-aphD*, *aphA3*, *cat*, *qacC*	PVL*; sea*, *sed*, *seh*, *sek*, *seq*	*agrIII; cap8; sak*, *scn; icaA/C/D*
	CC1-MRSA-[V/V_T_+*fusC*] (2)	t127 (2)	GM (1), K (1), TP (2), FD (2)	*ugpQ*, *mecA*, *fusC*, *ccrC*	*aacA-aphD*	*sea*, *seb*, *seh*, *sek*, *seq*	*argIII; cap8; sak*, *scn; icaA/C/D*
	CC1-MRSA-PseudoSCC*mec* [**class B mec**+*fusC*+*ccrA1B1*] (1)	t127 (1)	GM, K, FD	*ugpQ*, *mecA*, Delta *mecR1*, *fusC*, *ccrA/B-1*	*aacA-aphD*	*sea*, *seh*, *sek*, *seq*	*agrIII; cap8; sak*, *scn; icaA/C/D*
CC5	CC5-MRSA-[V/V_T_+*fusC*] (PVL^+^) (1)	t1588 (1)	FD (1)	*ugpQ*, *mecA*, *fusC*, *“ccrAA”*, *ccrC*	*fosB*	PVL; *sea*, *sec*, *sed*, *sej*, *sel*, *ser*, *egc	*agrII; cap5; sak*, *chp*, *scn; icaA/C/D*
CC6	CC6-MRSA-[IV+*fusC*+*ccrC*] (1)	t14700 (1)	C, FD	*ugpQ*, *mecA*, Delta *mecR1*, *fusC*, *ccrA/B-2*, *ccrC*, *dcs*	*fosB*	*Sea*	*agrI; cap8; sak*, *scn; icaA/C/D*
CC8	CC8-MRSA-[V/V_T_+*fusC*] (6)	t008 (5), t211 (1)	FD (6), CIP (6)	*ugpQ*, *mecA*, *fusC*, *“ccrAA”*, *ccrC*	*fosB*	*sea (N315)*, *seb*	*agrI; cap5; sak*, *scn; icaA/C/D*
CC22	CC22-MRSA-IV [tst1^+^ / PVL^+^] (12)	t005 (7), t309 (3), t223 (1), t10659 (1)	GM (9), K (8), E (8), CC (8), TP (11), CIP (9)	*ugpQ*, *mecA*, Delta *mecR1*, *ccrA/B-2*, *dcs*	*ermC*, *aacA-aphD*, *dfrS1*	PVL; *tst1*; *sec*, *sel*, *egc*	*agrI; cap5; sak*, *chp*, *scn; icaA/C/D*
	CC22-MRSA-[VI+*fusC*] (1)	t16578 (1)	FD	*ugpQ*, *mecA*, Delta *mecR1*, *fusC*, *ccrB-4*		*tst1; egc*	*agrI; cap5; sak*, *chp*, *scn; icaA/C/D*
**CC**	**Novel MRSA variants (N)**	**Spa types (N)**	**Antibiogram**	**SCC*mec*-associated markers**	**Antibiotic resistance genes**	**Toxin genes**	**Miscellaneous**
CC30	CC30-MRSA-[VI+*fusC*] (PVL+) (12)	t018 (6), t012 (1), t021 (4), t318 (1)	K (2), E (3), CC (3), TE (4), TP (4), FD (11), CIP (1)	*ugpQ*, *mecA*, Delta *mecR1*, *fusC*, *ccr(A)/B-4*, *dcs*	*ermC*, *linA*, *aadD*, *tetK*, *dfrS1*, *fosB*	PVL; *tst1; sea*, *egc*	*agrIII; cap8; sak*, *chp*, *scn; icaA/C/D*
	CC30-MRSA-[VI+*fusC*] (3)	t018 (1), t021 (1), t253 (1)	TE (1), TP (1), FD (2)	*ugpQ*, *mecA*, Delta *mecR1*, *fusC*, *ccr(A)/B-4*, *dcs*	*aadD*, *tetK*, *fosB*	*tst1*; *sea*, *egc*	*agrIII; cap8; sak*, *chp*, *scn; icaA/C/D*
CC45	CC45-MRSA-[VI+*fusC*] (2)	t362 (2)	FD (2)	*ugpQ*, *mecA*, Delta *mecR1*, *fusC*, *ccrA/B-4*, *dcs*		*egc*	*agrI; cap8; sak*, *chp*, *scn; icaA/C/D*
CC88	CC88-MRSA-[IV+*fusC*] (2)	t786 (1), t2622 (1)	TP (1), FD (2), TE (2)	*ugpQ*, *mecA*, Delta *mecR1*, *fusC*, *ccrA/B-2*, *(dcs*, *ccrB4)*	*dfrS1*, *tetK*	*sea (N315)*	*agrIII*, *cap8; sak*, *chp*, *scn;icaA/C/D*
CC97	CC97-MRSA-[V/VT+*fusC*+*ccrA2B2*] (1)	t2297 (1)	FD	*ugpQ*, *mecA*, *fusC*, *ccrA/B-2*, *“ccrAA”*, *ccrC*			*agrI; cap5; sak*, *scn; icaA/C/D*
	CC97-MRSA-[VI+*fusC*] (1)	t359 (1)	TP, FD	*ugpQ*, *mecA*, Delta *mecR1*, *fusC*, *ccrA/B-4*	*dfrS1*		*agrI; cap5; sak*, *scn; icaA/C/D*
CC121	CC121-MRSA-[V/V_T_+*fusC*] (PVL^+^) (5)	t314 (4), t1991 (1)	GM (5), K (5), TE (4), FD (5), CIP (1)	*ugpQ*, *mecA*, *fusC*, *“ccrAA”*, *ccrC*	*aacA-aphD*, *tetK*, *fosB*	PVL; *seb*, *egc*	*agrIV; cap8; sak*, *scn; icaA/C/D*
	CC121-MRSA-[V/V_T_+*fusC*+*ccrB4*] (PVL+) (1)	t314 (1)	FD	*ugpQ*, *mecA*, *fusC*, *“ccrAA”*, *ccrC*, *ccrB-4*	*fusC*, *fosB*	PVL; *seb*, *egc*	*agrIV; cap8; sak*, *scn; icaA/C/D*
CC152	CC152-MRSA-[V+*fusC*] (PVL+) (5)	t355, t4019, t11206	GM (4), K (4), TE (4), FD (5)	*ugpQ*, *mecA*, *fusC*, *“ccrAA”*, *ccrC*	*aacA-aphD*, *tetK*	PVL	*agrI; cap5; sak/scn; icaA/D; edinB*
**CC**	**Novel MRSA variants (N)**	**Spa types (N)**	**Antibiogram**	**SCC*mec*-associated markers**	**Antibiotic resistance genes**	**Toxin genes**	**Miscellaneous**
CC152	CC152-MRSA-[VI+*fusC*] (PVL+) (1)	t4019	TP, TE, FD	*ugpQ*, *mecA*, Delta *mecR1*, *fusC*, *ccrB-4*	*dfrS1*, *tetK*	PVL	*agrI; cap5; sak/scn; icaA/D; edinB*
CC361	CC361-MRSA-[V/V_T_+*fusC*] (32)	t3841 (28), t1309 (1), t15778 (1), t3175 (1), t16901 (1)	GM (4), K (18), E (16), CC (3), C (1), TP (30), TE (2), FD (28), CIP (30)	*ugpQ*, *mecA*, *fusC*, *“ccrAA”*, *ccrC*	*msr(A)*, *mphC*, *ermC*, *aphA3*, *sat*, *dfrS1*, *tetK*, *fosB*, *qacC*	*sed*, *egc*	*agrI; cap8; sak*, *scn; icaA/C/D*
CC913	CC913-MRSA-[VI+*fusC*] (3)	t991 (3)	E (3), CC (3), TP (3), FD (3)	*ugpQ*, *mecA*, Delta *mecR1*, *fusC*, *ccrB-4*, *(dcs)*	*ermC*, *dfrS1*, *fusC*	*etA*, *etD*	*agrII; cap8; sak/chp/scn; icaA/C/D; edinB*
CC1153	CC1153-MRSA-[I+*fusC*] PVL+ (1)	t504	FD	*ugpQ*, *mecA*, *Delta mecR1*, *fusC*, *ccrA/B-1*		PVL	*agrII; cap5; sak*, *scn; icaA/C/D*
Singleton	ST2867-MRSA-V/V_T_ (1)	t148		*ugpQ*, *mecA*, *ccrC*	*fosB*		*agrII; cap5; sak/scn; icaA/C/D; edinB*

**Abbreviations:** C, Chloramphenicol; FD, fusidic acid; GM, gentamicin; CIP, ciprofloxacin; CC, clindamycin; E, erythromycin; K, kanamycin; TE, tetracycline; TP, trimethoprim.

*egc (*seg*, *sei*, *selm*, *seln*, *selo*, *selu*).

ND-not determined

### CC1

Eleven isolates constituted three novel variants of CC1-MRSA comprising CC1-MRSA-[V/V_T_+*fusC*] (PVL^+^) (8 isolates), CC1-MRSA-[V/V_T_+*fusC*] (2 isolates), and CC1-MRSA-PseudoSCC*mec* [class B mec+*fusC*+*ccrA1B1*] (1 isolate). Isolates of the CC1-MRSA-[V/V_T_+*fusC*] (PVL^+^) variant belonged to spa types t127 (2 isolates) and t2207 (6 isolates). Normally, CC1 isolates carry SCC*mec* IV/V elements plus *fusC*+*ccrA1B1* [7,14]. However, these new variants lacked *ccrA1B1*. Besides the abscence of *ccrA1B1* in these isolates, isolates with *spa* type t2207 or those with pseudoSCC*mec* (a variant of SCC*mec* that lost recombinase (*ccr*) genes) [20] as in CC1-MRSA-PseudoSCC*mec* [class B mec+*fusC*+*ccrA1B1*] have not been previously reported in Kuwait. The CC1-MRSA-[V/V_T_+*fusC*], and CC1-MRSA-[V/V_T_+*fusC*] (PVL^+^) isolates carried the SCC*mec* subtype V_T_, a composite genetic element comprising SCC*mec* and fusidic acid resistance gene *fusC*, which is novel in Kuwait. The majority of the CC1 isolates were resistant to fusidic acid, kanamycin, erythromycin and clindamycin mediated by *fusC*, *aacA-aphD* and *erm(C)* respectively. However, the isolates belonged to *agr*III, *cap*5 and harbored enterotoxin genes, *seh*, *sek* and *seq* previously associated with CC1-MRSA isolates [7].

### CC5

The novel variant of CC5-MRSA genotype, CC5-MRSA-[V/V_T_+*fusC*], carried genes for both TSST1 and PVL (Table 1). The isolate also carried a composite genetic element, SCC*mec* V_T_ plus fusidic acid resistance gene, *fusC*. It belonged to spa type t1588 and was only resistant to fusidic acid in contrast to the CC5-SCC*mec* V-t688 isolate previously reported in Kuwait that was resistant to tetracycline mediated by *tet(K)* and *tet(M)* [14]. However, the isolate carried *agr*II and *cap*5 which are typical of CC5-MRSA isolates [7].

### CC6

The single variant of CC6-MRSA, CC6-MRSA-[IV+*fusC*+*ccrC*], carried *ccrA2B2* and an additional ccr allotype, *ccrC*. It belonged to a novel spa type, t14700, and carried *agr*I, *cap*8. It was positive for enterotoxin gene, *sea* and the fusidic acid resistance determinant *fusC*.

### CC8

The six isolates belonging to the CC8-MRSA [V/V_T_ +*fusC*] variant were unique because they carried a composite SCC*mec*V+*fusC* element which is not common in this lineage. These isolates belonged to spa types t008 (5 isolates) and t211 (1 isolate), harbored *sea* and *seb*, and were resistant to fusidic acid (Table 1) and differed from the CC8-V-MRSA isolate that was obtained in 2010 in a Kuwait hospital [14]. The 2010 CC8-V-MRSA was resistant to erythromycin, gentamicin and kanamycin, belonged to spa type, t064 and harbored *agr*I, *cap*5, and enterotoxins genes *sea*, *seb*, *sek*, *seq*.

### CC22

Twelve of the 13 variant isolates of CC22-MRSA consisted of isolates belonging to spa types t005 (7 isolates), t223 (1 isolate), t309 (3 isolate) and t10659 (1 isolate) were identified as CC22-MRSA-IV (tst1^+^ / PVL^+^) (Table 1). These isolates were unique because they carried the unusual combination of PVL and TSST1. In addition, an isolate identified as CC22-MRSA-[VI+*fusC*] belonged to spa type t16578 and carried the SCC*mec* VI genetic element not previously identified in CC22-MRSA. This isolate was resistant to fusidic acid, was PVL-negative but was positive for *tst*1, and carried egc, *agr*I and *cap*5.

### CC30

Fifteen isolates belonged to CC30-MRSA-[VI+*fusC*] (PVL^+^) (12 isolates) and CC30-MRSA-[VI+*fusC*] (3 isolates). These variants harbored a unique composite genetic element consisting of SCC*mec* VI, which is unusual in this lineage, and *fusC*. The CC30-MRSA-[VI+*fusC*] (PVL^+^) isolates belonged to spa types t018 (6 isolates), t012 (1 isolate), t021 (4 isolate), and t318 (1 isolate). Twelve isolates were positive for PVL, *tst1*and enterotoxins, *sea* and ***egc***, *agr*III and *cap*8. All PVL-positive isolates carried *fusC* that mediates fusidic acid resistance but expressed varied resistance to erythromycin, clindamycin, kanamycin and trimethoprim mediated by *erm(C)*, *aadD* and *dfrS1*, respectively (Table 1). The CC30-MRSA-[VI+*fusC*]—PVL-negative isolates belonged to spa types t018 (1 isolate), t021 (1 isolate) and t253 (1 isolate) and carried *aadD*, *tet(K)* and *fusC* (Table 1).

### CC45

The CC45-MRSA variant genotype, C45-MRSA-[VI+ *fusC*], was identified in two isolates belonging to spa type t362. The isolates carried a composite element, SCC*mec* VI + fus, which is rare in this lineage.

### CC88

The two CC88 -MRSA variants belonged to spa types t786 and t2622. Both isolates belonged to genotype, CC88-MRSA-[IV+*fusC*]. These isolates carried a composite genetic element consisting of SCC*mec* IV and fusidic acid resistance gene *fusC*. Both isolates were resistant to tetracycline mediated by *tet(K)* but differed in the carriage of enterotoxin genes. While the t2622 isolate carried *sea*, the t786 isolate lacked enterotoxin genes. In addition, the t786 isolate was resistant to trimethoprim mediated by *dfrS1*. Both carried *agr*III and *cap*8.

### CC97

Two CC97-MRSA variants belonging to spa types t2297 and t359 were identified as CC97-MRSA-[V/V_T_+*fusC*+*ccrA2B2*] and CC97-MRSA-[VI+*fusC*] respectively (Table 1). The CC97-MRSA- [V/V_T_+*fusC*+*ccrA2B2*]-t2297 isolate may harbor a new SCC*mec* element because it carries an additional *ccrA2B2* element which is usually found in SCC*mec* IV isolates [7]. It was negative for genes encoding PVL, TSST1 and enterotoxins, but was positive for *agr*I, *cap*5 and to the fusidic acid resistance determinant, *fusC*. The t359 isolate was unique because it carried SCC*mec* VI which is novel in this clonal complex. The isolate lacked genes for PVL, TSST1 and enterotoxins but carried *fusC* and *dfrS1* which mediate resistance to fusidic acid and trimethoprim respectively.

### CC121

Six isolates were identified as variants of CC121-MRSA. Five of the isolates were identified as CC121-MRSA-V/V_T_+*fusC* [PVL^+^] (Table 1). These isolates carried a unique composite genetic element consisting of SCC*mec* V and the fusidic acid resistance gene, *fusC* and differed from the CC121-IV-MRSA isolates reported previously in Kuwait which were susceptible to fusidic acid [14]. The sixth isolate was identified as CC121-MRSA-[V/V_T_+*fusC*+*ccrB4*] (PVL^+^). This isolate was unique because it carried an additional *ccrB4* but *ccrA4* was not detected. The *ccrA4B4* allotype is usually found in isolates carrying SCC*mec* VI or VIII [21]. It was resistant to fusidic acid mediated by *fusC* and contained *seb* and *egc*. All six isolates were positive for *agr*IV and *cap*8.

### CC152

Six isolates were identified as CC152-MRSA-[V+*fusC*] (PVL+) (5 isolates) and CC152-MRSA-[VI+*fusC*] (PVL+) (1 isolate). The CC152-MRSA-[V+*fusC*] (PVL+) isolates carried a composite element comprising SCC*mec* V and the fusidic acid resistance gene *fusC*. These variants belonged to spa types t355, t4019 and t11206 (Table 1). All six isolates harbored genes for PVL but lacked genes for enterotoxins. The isolates varied in their carriage of antibiotic resistance genes, *aacA-aphD*, *fusC*, *tet(K)* and *dfrS1*. One isolate lacked *tet(K)* while another isolate carried *tet(K)* but lacked *aacA-aphD*. The CC152-MRSA-[VI+*fusC*] (PVL+) harbored SCC*mec* VI genetic element which was not common in CC152-MRSA isolates. The t4019 isolate harbored *dfrS1*, *fusC*, *tet(K)*, that mediate resistance to trimethoprim, fusidic acid and tetracycline respectively. All six CC152-MRSA variants belonged to *agr*I and *cap*5.

### CC361

Thirty-two isolates were identified as variants of CC361-MRSA. All 32 isolates, identified as CC361-MRSA-[V/V_T_+*fusC*], harbored a composite genetic element consisting of SCC*mec* V_T_ and the fusidic acid resistance gene *fusC*. The isolates belonged to spa types, t3841 (28 isolates), t1309 (1 isolate), t15778 (1 isolate), t3175 (1 isolate), and t16901 (1 isolate). All isolates carried *agr*I and *cap*8 and expressed multiresistance to antibiotics (Table 1).

### CC913

Three isolates were identified as variants of CC913-MRSA. All three isolates, identified as CC913-MRSA-[VI+*fusC*], belonged to spa type t991. The isolates were unique because they carried SCC*mec* VI which is new in this lineage. All three isolates lacked genes for PVL, TSST1and enterotoxins, but carried *agr*II, *cap*8, *etA* and *etD* encoding exfoliative toxins A and D respectively. All isolates carried *erm(C)*, *dfrS1* and *fusC* encoding resistance to erythromycin and clindamycin, trimethoprim, and fusidic acid respectively. One isolate carried an additional determinant, *vgaA*, that mediates streptogramin-A resistance (Table 1).

### CC1153

The CC1153/t504 isolate identified in this study, CC1153-MRSA [I+*fusC*] PVL^+^ was unique because it carried a novel composite genetic element composed of SCC*mec* I and a SCC*fus*/*ccrA1B1* element. It lacked enterotoxin genes but was positive for PVL and *fusC* that mediates fusidic acid resistance.

### ST2867

One isolate was identified as ST2867-MRSA-V/V_T_. The variant carried the SCC*mec* V variant V_T_ which was not reported in this singleton before.The isolate lacked genes for PVL,TSST1 and enterotoxins but carried *agr*II and *cap*5 (Table 1).

## Discussion

The application of molecular typing and DNA microarray analysis facilitated the discovery of novel MRSA variants within existing clonal complexes in Kuwait hospitals. Of the 102 MRSA isolates, 88 carried composite genetic elements comprising SCC*mec* and fusidic acid resistance gene *fusC* (Table 1). The carriage of these elements may be beneficial for the survival of the strains. The co-residence of *fusC* and SCC*mec* genetic element may ensure the stability of fusidic acid resistance and enhance its spread. Although *fusC*-mediated fusidic acid resistance has previously been reported in ST239-III-MRSA clone in Kuwait [14] the presence of *fusC* in isolates belonging to different genetic backgrounds suggests that it was acquired independently by the different clones probably involving a common bacteriophage. It is also possible that the composite SCC*mec* IV+*fusC* and SCC*mec*V+*fusC* elements have started wandering across different lineages that are favored by certain selective pressures such as the use of over-the-counter fucidin ointments, or a drive to replace mupirocin use with fucidin as currently observed in New Zealand [22, 23]. While investigating fusidic acid-resistant MRSA in the UK, Ellington *et al*., [24] observed that the fusidic acid resistance determiminant, *fusC*, was located on multiple SCC*mec* elements including chimeric cassette elements that conferred resistance to beta-lactams and fusidic acid. Therefore, the composite elements involving SCC*mec* and *fusC* oberved in this study may represent chimeric elements similar to the report by Ellington et al [24]. The emergence of different chimeric genetic elements involving SCC*mec*, antibiotic resistance and virulence genes in MRSA may signal new adaptive mechanisms for their survival in the presence of antibiotics.

Curiously, the majority (32 of 102) belonged to CC361-MRSA which have been observed in small numbers in previous reports from human [14, 25, 26] and animal [7, 27, 28] sources. Furthermore, 28 of the 32 isolates belonged to a single spa type, t3841,which were isolated from wound, nasal, respiratory, ear, vaginal, groins and axilla in seven hospitals signifying the capacity of the clone to spread, colonize as well as cause infections in humans. However, the apparent high prevalence of CC361-MRSA in this study was due to the detection of a single CC361-MRSA-t3841 clone in different hospitals. Continuous surveillance is needed to monitor future expansion of this clone in the country.

The study also revealed the presence of probable novel SCC*mec* (3 isolates) and pseudo SCC*mec* (1 isolate) elements (Table 1). DNA Microarray analysis revealed the presence of either *ccrA2B2* or *ccrC* elements in isolates belonging to CC6-MRSA-[IV+*fusC*+*ccrC*]and CC97-MRSA-[V/V_T_+*fusC*+*ccrA2B2*]. Additionally, an isolate identified as CC121-MRSA-[V/V_T_+*fusC*+*ccrB4*] (PVL+) contained *ccrB*4 that is not usually associated with SCC*mec* type V suggesting that this may be a new SCC*mec* element. Also, an isolate with a novel genotype, CC1-MRSA-PseudoSCC*mec* [class B mec+*fusC*+*ccrA1B1*]/t127, was detected among the CC1-MRSA (Table 1). The *ccrC* gene is usually found in isolates carrying SCC*mec* V and the *ccrA2B2*, has been associated with isolates carrying SCC*mec* II or SCC*mec* IV [7]. The carriage of these novel elements in non-traditional backgrounds may represent the emergence of novel SCC*mec* elements. These composite genetic elements probably evolved by recombinations between genetic elements native to the host and acquired foreign DNA as has been reported in other MRSA backgrounds [20]. Previously, *ccrA4B4* which shared 100% identity to similar elements in *S*. *epidermidis* was detected in ST8-MRSA-t190 isolates obtained in Irish hospitals [29] suggesting that *ccrA4B4* was acquired from *S*. *epidermidis* [30]. Additionally, a novel pseudo SCC*mec* element carrying *mecA* with a novel *mec* class region and fusidic acid resistance gene (*fusC*) was detected in ST779-MRSA also in Irish hospitals [31]. The genetic backgrounds of our isolates are different from those isolated in Irish [29] and Indian [32] hospitals suggesting that these events are probably more widespread than currently appreciated and highlights the value of DNA Microarray in detecting these events. However, Whole genome sequencing of these isolates will be required to clearly understand the organization of these novel composite elements. SCC*mec*- associated genes.

This study also revealed the carriage of an unusual combinations of genes encoding TSST1 and PVL in the same cell in isolates belonging to CC22-MRSA and CC30-MRSA (Table 1). Previously, some CC22-MRSA subtypes had either carried genes for PVL as in ST22-IV-t852/t790 [33, 34] or *tst1* as in the Middle East variant of EMRSA-15), CC22-IV-MRSA/ tst1+] [33, 35, 36]. However, recently, Khairalla *et al*., [37] reported CC22-IV-MRSA isolates obtained from three healthcare workers in a dental practice in Egypt that carries both *tst1* and PVL suggesting that this may be an emerging trait in CC22-IV-MRSA.Their three isolates belonged to spa types, t14339 (2 isolates) and t8506 (1 isolate) and were resistant to gentamicin and clindamycin. In contrast, the isolates in this study belonged to spa types, t005, t309 and t223 but were also resistant to gentamicin and clindamycin. It is not clear whether the carriage of both determinants act synergistically to elevate the virulence capacity of these isolates.

This is the first report of MRSA with SCC*mec* V subtype V_T_ in Kuwait. Isolates that carrry SCC*mec* V_T_ were first reported in 2005 in Taiwan by Boyle-Vavra *et al*., [38]. Whereas, SCC*mec* V element harbor ccr complex class C1 and mec complex class C2 [36], SCC*mec* V_T_ contains a *ccrC2* which is a *ccrC* recombinase gene variant and *mec* complex C2. All of the Taiwaness SCC*mec* V_T_ strains belonged to ST59 [38], In contrast, the isolates in this study with SCC*mec* V_T_ element belonged to different lineages including CC1, CC5, CC8, CC97, CC121 and CC361. Similarly, MRSA isolates carrying SCC*mec* V_T _have been reported in different clonal complexes including CC7, CC8, CC30, CC45, CC59, CC152, and CC398 in Germany [39] suggesting the independent acquisition of SCC*mec* V_T_.

The study further revealed the occurence of SCC*mec* type I and type VI in lineages such as CC22, CC30, CC45, CC97, CC152, CC913, and CC1153 that usually carry SCC*mec* types IV or type V [8, 21]. Reports of CC1153-MRSA are rare in the literature, and a previously reported isolate in Kuwait carried SCC*mec* V element and was susceptible to fusidic acid [40]. Therefore the carriage of SCC*mec* type 1 and *fusC* in an isolate of CC1153-MRSA in this study represents a novel development.

The SCC*mec* type VI element was detected in 15 CC30-MRSA and three CC913-MRSA isolates. Until now, only two isolates of CC30-MRSA-[VI+*fusC*] (PVL+) was previously reported in Saudi Arabia [41]. The 15 CC30-MRSA-[VI+*fusC*] (PVL+) isolates belonged to five spa types including t012, t018, t021 that are usually associated with CC30-MRSA suggesting that the compositive genetic element was recently acquired by CC30-MRSA. The other spa types, t253 and t318 represent sporadic isolates. Although epidemiologic relationship could not be established between the isolates from Kuwait and Saudi Arabia, their identification in 15 isolates in this study may signal the expansion of this lineage in the region.

CC152 isolates are rare and those reported earlier carried SCC*mec* V [7], which is different from the SCC*mec* VI in the isolate in this study. In addition, the detection of spa type t362 in SCC*mec* VI that was not detected previously in CC45 isolates obtained in Kuwait [14] indicates the emergence of new CC45 variants in Kuwait hospitals. The emergence of unusual SCC*mec* types in genetic backgounds that they are not normally associated with may cause difficulty in classifying these MRSA isolates on the basis of their SCC*mec* elements.

The main limitation of this study is the absence of whole genome sequencing data of the isolates to further analyze these variant strains. Whole genome sequencing will be required to understand the orgnization of the SCC*mec* encoding genes which may help identify new SCC*mec* elements.

## Conclusion

This study has revealed the emergence of novel variants of known MRSA genotypes in Kuwait hospitals. The new isolates all belonged to CA-MRSA genotypes and highlights the growing diversity of CA-MRSA strains globally [1, 3, 7, 41]. It also points to a shift from an era where a small number of highly prevalent epidemic strains with small numbers of SCC*mec* elements to a high number of very diverse strains and very diverse SCC*mec* elements that both recently evolved or continues to evolve. While this makes molecular typing more interesting, it spells trouble for infection control. Therefore, conducting molecular surveillance is important at regular intervals to prevent the establishment and trasnmission of these strains in Kuwait healthcare facilities.
